# Injectable, pore-forming, self-healing, and adhesive hyaluronan hydrogels for soft tissue engineering applications

**DOI:** 10.1038/s41598-023-41468-9

**Published:** 2023-08-31

**Authors:** Sara Nejati, Luc Mongeau

**Affiliations:** Department of Mechanical Engineering, McGill University, Montreal, Canada

**Keywords:** Chemistry, Engineering

## Abstract

Most existing injectable hydrogels are non-porous, thereby lacking a microporous structure to promote cell ingrowth. Also, most hydrogels do not effectively adhere to the host tissue. The present study describes an injectable double network hydrogel formed by combining two hyaluronic acid (HA) derivatives, namely dopamine grafted HA (DAHA) and methacrylated HA (HAMA). These constituents instantly form a physically crosslinked network through Fe^3+^-dopamine coordination, and confer fast gelation, pore formation, and self-healing properties to the hydrogel. Photocroslinked upon UV exposure, HAMA forms a chemically crosslinked network, thereby improving mechanical and degradation properties. The adhesive properties of this hydrogel are attributed to the presence of dopamine groups, inspired by mussel creatures. Proper modification of HA chains was confirmed by NMR spectroscopy. The physical, mechanical, rheological, and biological properties of the new hydrogels were quantified in wet laboratory conditions. The results revealed that the DAHA/HAMA hydrogel rapidly forms a self-healing microporous adhesive scaffold with a 26.9 µm pore size, 29.4 kPa compressive modulus, and 12.8 kPa adhesion strength in under 6 s. These findings suggest that the new hydrogel is a promising candidate for in situ repair of soft tissues, particularly mechanically dynamic ones such as the vocal folds, cartilage, and dermis.

## Introduction

Injectable hydrogels are biomaterials for regenerative medicine applications with a minimally invasive nature and the ability to refill irregularly shaped defects^1^. Hydrogels must meet many material design requirements to be considered suitable for in situ injection purposes. A fast gelation is needed to prevent the pregel from leaking into surrounding areas. A microporous structure is desirable to promote cell ingrowth and nutrient transportation. Strong adhesion to the tissue is needed in order to prevent detachment from the target tissue.

Naturally derived polymers are advantageous to this end because of their excellent biocompatibility and biodegradability. Hydrogels derived from naturally occurring polysaccharides mimic many features of the extracellular matrix (ECM). They have displayed the potential to direct the migration and proliferation of encapsulated cells during tissue regeneration^2–4^.

**Figure 1:**
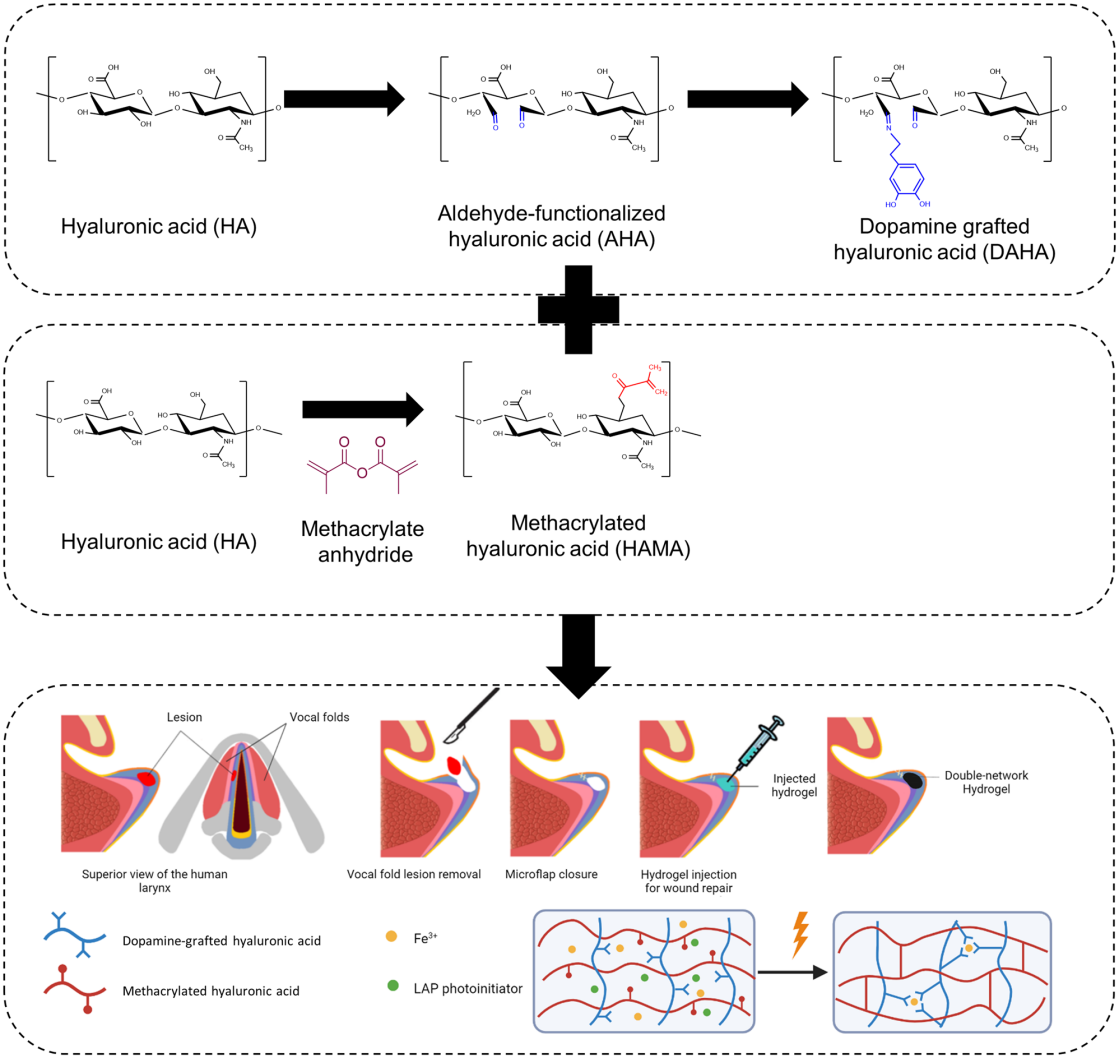
Schematic illustration of (**a**) dopamine-grafted hyaluronic acid (DAHA) synthesis, (**b**) methacrylated hyaluronic acid (HAMA) synthesis, and (**c**) the in situ injectable double network hydrogel for vocal folds tissue engineering.

Hyaluronic acid (HA) is a naturally non-sulphated polysaccharide consisting of multiple repeating disaccharide units of *N*-acetyl-D-glucosamine and D-glucuronic acid. In the ECM, hyaluronic acid constitutes the backbone of glycosaminoglycan (GAG) superstructure complexes; it contributes to tissue hydration, nutrient diffusion, proteoglycan organization, and cell differentiation^5–7^. Hyaluronic acid-based hydrogels also have a few noteworthy limitations, which have hampered somewhat their potential applications. Such limitations include fast degradation, the paucity of cell adhesion sites, weak tissue adhesion, high degrees of swelling, and its rigid chain conformation causing its brittleness. Modifications of HA chains are needed to address existing limitations and further improve HA applicability for in situ injection applications. A schematic of the hydrogel concept used in the present study is shown in Fig. 1 and the rationale behind applied modifications are elaborated below.

The intrinsically rigid chain conformation of HA causes the hydrogels to be naturally brittle, unable to retain their structural integrity under mechanical challenges. This is particularly problematic for mechanically dynamic tissues^8^. It has been shown that the partial oxidation of HA with periodate can address this problem. Hydrogels obtained from HA with an open-ring structure are more elastic than unmodified HA- based hydrogels^9,10^.

Good adhesion is needed for hydrogels to strongly adhere to the host tissue. Mimetic mechanical properties are also highly desirable for tissue repair. As adhesive injectable hydrogels may enhance the minimally invasive delivery of therapeutic agents, they can better retain the delivered agents at host tissue locations and prevent debonding from the targeted location. This property is very important for in situ injection in vocal folds as insufficient tissue adhesion and subsequent hydrogel detachment may cause airway occlusion and lead to patient death.

In a marine environment, specific proteins are secreted by mussels and used as a bioglue for surface adhesion. These mussel proteins present an unusual amino acid 3, 4-dihydroxyphenylalanine known as dopamine. The outstanding adhesive properties of these materials in the harsh sea conditions have been attributed to the catechol groups present in dopamine^11,12^. Inspired by these adhesive proteins and using the aldehyde groups presented on HA chains after oxidation, dopamine-modified hyaluronic acid was prepared. The decoration of HA chains with dopamine groups can address the HA’s poor cell adhesion property^13,14^. Anchorage dependant cells (e.g., fibroblasts) must attach to a substrate to survive, grow, divide and function properly. Poor cell adhesion and spreading may cause cells to lose their healthy morphology and behavior. The availability of cellular binding sites for cells to attach largely depends on the chemical composition of the hydrogel. Unmodified HA lacks binding sites and, as a result, does not support cell adhesion and spreading. The addition of dopamine groups onto its chains offers a pragmatic solution to this problem^15,16^.

Most injectable hydrogels in use or under development are not perfusable due to their nanoporous structures, hampering their effectiveness for tissue engineering purposes^17–19^. To promote tissue or organ regeneration, the porous scaffold must mimic a native extracellular matrix, onto which cells attach, proliferate, migrate and function. Porous scaffolds facilitate the rapid transport of oxygen and nutrients, as well as the trafficking of native or transplanted cells in the scaffolds. These micro pores can be formed either prior to injection using techniques such as cryogelation, lyophilization, or 3D printing, or in situ after injection of the pregel using porogen, leachable particles, granular particles, polymer degradation, or self-assembly. In situ pore formation is preferrable to preformed porous structures because preformation requires lengthy fabrication processes as well as prior knowledge of the defect shape. Preformation also increases the risk of collapse during injection^8,20^. Hence, an in situ pore formation approach was followed in this study. Iron was incorporated to induce Fe^3+^-dopamine coordination complexation to trigger the instantaneous formation of a microporous network. Dopamine contains catechol, a functional group which exhibits chelating properties towards metal ions, including Fe^3+^. Iron has a high affinity for oxygen donor atoms, and catechol's hydroxyl groups can act as donors and effectively coordinate with iron ions through coordination bonds, forming complexes known as chelates. The chelating properties of catechol have been extensively studied and exploited in various fields, including biomaterials, drug delivery systems, and metal ion sensing^21^. This approach has the additional benefit of making the gel self-healing^15,22,23^. Self-healing hydrogels may break down under stress but recover their initial integrity following the removal of applied forces.

A secondary crosslinking mechanism is needed to enhance mechanical properties, reduce degradation rates, and reduce swelling. In the present study, HA chains were modified with methacrylate groups. The photopolymers (HAMA) were cured rapidly using UV exposure. The spontaneously formed physical network could further be strengthen and chemically crosslinked through UV exposure^24,25^.

The proposed hydrogel has many different potential applications, for example, cartilage, skin, and gut. The targeted application in the current study is vocal fold repair. Vocal folds are soft laryngeal connective tissues with distinct layered structures. Vocal fold defects caused by various environmental factors and pathological conditions remain among the foremost therapeutic challenges that detrimentally affect the daily lives of suffering individuals. It is estimated that up to 9% of the general population suffer from a voice disorder at one point over their lifetime. The repair of damaged vocal folds using injectable hydrogels is an attractive approach because they require only minimally invasive procedures and can fill irregular defects^26,27^. Hyaluronic acid is the most abundant glycosaminoglycan in the vocal folds and plays many critical roles in this tissue^28,29^. It acts as a space filler, shock absorber, and tissue damper, which are particularly useful properties as vocal folds which are under constant vibration. HA regulates vocal folds’ hydration through modulation of the tissue viscosity and osmosis. Moreover, cell surface receptors CD44 and receptor for hyaluronan-mediated motility bind to HA and trigger specific signaling pathways which regulate inflammation, cell motility, and cell growth, leading to scarless would healing. The bioactivity of HA in promoting wound healing combined with its role in maintaining vocal fold hydration and biomechanics make HA an attractive building block for scaffolds of tissue engineered vocal folds^30–32^. Hence, the developed double network hydrogel would be a promising candidate for VF tissue regeneration.

## Results and discussion

### Chemical characterizations

**Figure 2:**
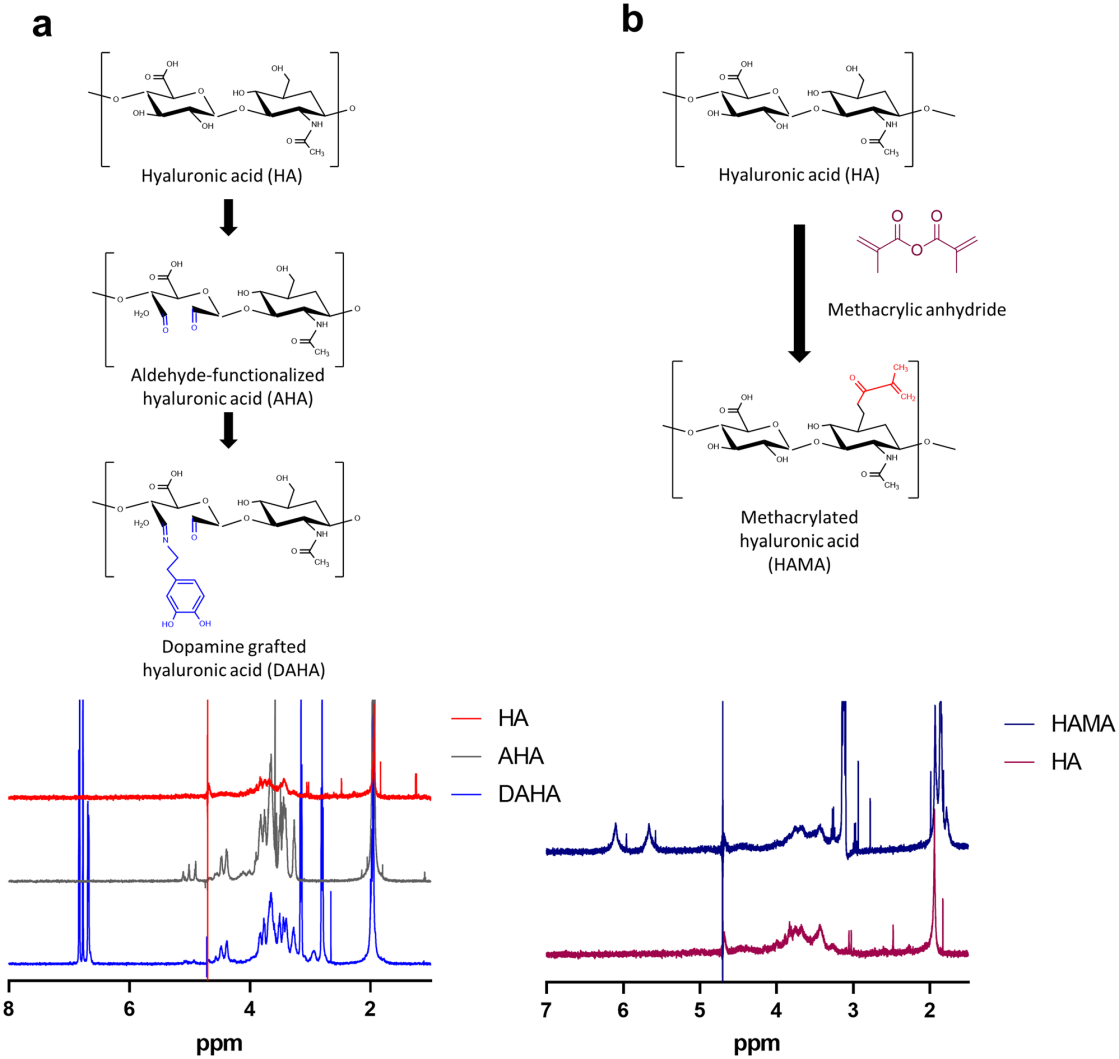
Chemical characterization of hyaluronic acid and its derivatives prepared in D_2_O. (**a**) Chemical structures and ^1^H NMR spectra of HA (top), AHA (middle) and DAHA (bottom) confirming the presence of aldehyde groups on AHA and dopamine groups on DAHA. (**b**) Chemical structures and ^1^H NMR spectra of HA (bottom) and HAMA (top) confirming the successful methacrylation of HA.

The synthesis of HAMA photopolymers and the degree of methacrylation were verified and quantified by 1H NMR analysis, as shown in Fig. 2. The NMR spectrum of HA revealed the presence of HA methyl protons at 1.9 ppm. After methacrylation, the protons in methacrylate vinyl groups appeared at 5.6 ppm and 6.1 ppm. The MA methyl protons were confirmed at 1.8 ppm. The degree of methacrylation was determined as the ratio of the integral of the HA methyl proton peak (at ~ 1.9 ppm) to that of the protons of MA (at ~ 5.6 ppm and ~ 6 ppm). The degree of methacrylation was calculated as 91%, which represents nearly one methacrylate group per disaccharide unit of HA^33^.

Dopamine-modified HA (DAHA) was synthesized by grafting dopamine, a catecholic monomer, onto aldehyde-modified HA via a Schiff base reaction. The successful conjugation of dopamine into HA molecules was further confirmed by 1H NMR analysis (Fig. 2). The presence of catechol aromatic proton peaks at *δ* ∼ 7 ppm and catechol methylene-proton peaks at *δ* 3.1 and 2.8 ppm, thereby demonstrating proper conjugation. The degree of substitution (DS) of catechol groups was then determined from the spectrum of DAHA according to the equation DS = 3 × A2.8/(2 × A1.9), where A2.8 and A1.9 are the integration of peak area at *δ* = 2.8 (methylene of catechol moieties) and *δ* = 1.9 (methyl of HA), respectively. The DS was calculated to be approximately 36%, which is much greater than values found in most previous studies. Previous attempts at grafting dopamine onto HA chains using EDC/NHS chemistry have yielded a degree of substitution of 10–12%^34,35^.

### Physical characterizations

#### Morphology

**Figure 3:**
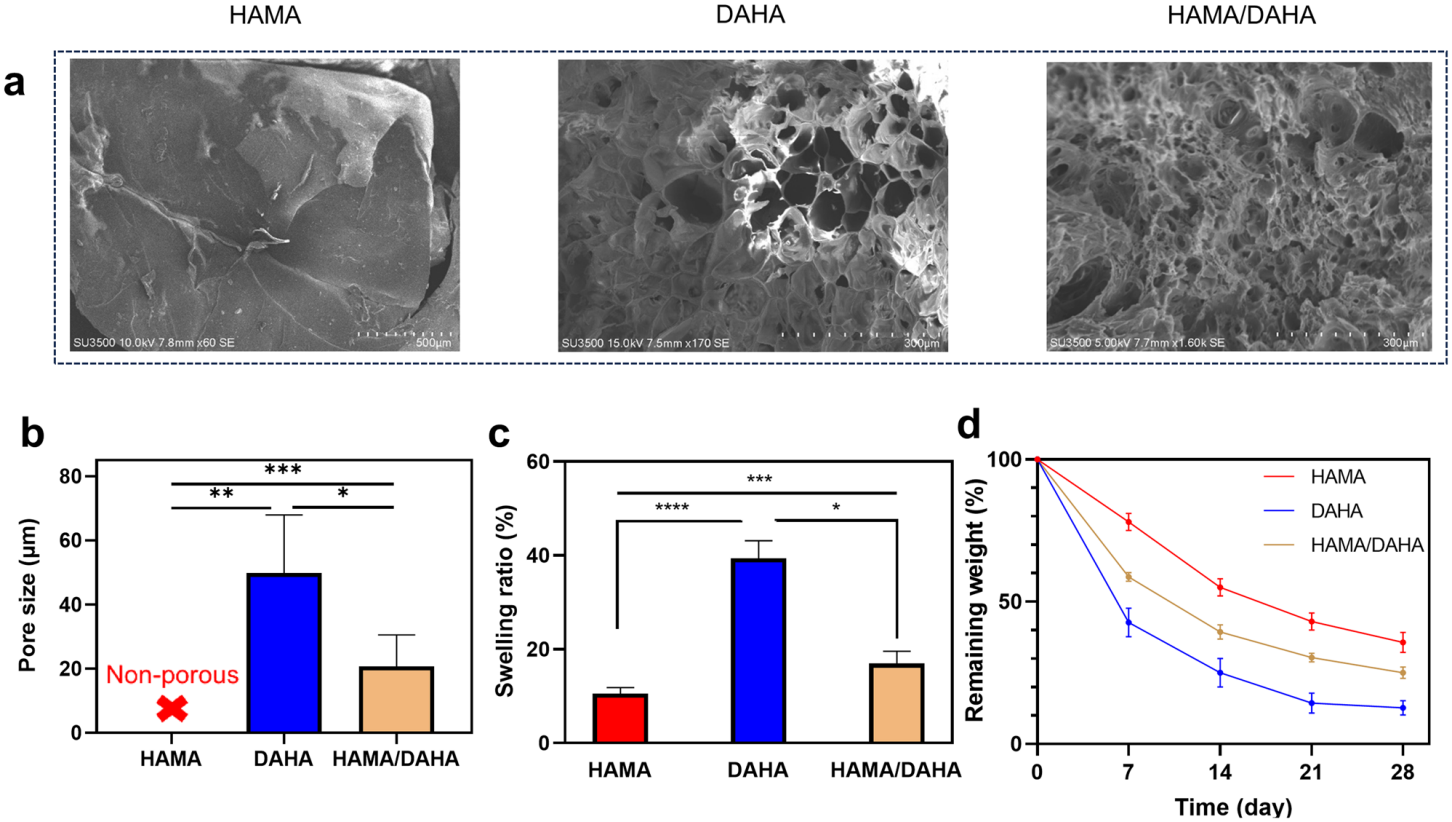
Physical properties of hydrogels. (**a**) SEM images showing the morphology and microstructure of samples. (**b**) Pore size of hydrogels. (**c**) The swelling ratio of samples after 48 h incubation in PBS. (**d**) degradation profile of hydrogels immersed in hyaluronidase solution over a period of 4 weeks. Sample size, *N* = 3; ** represents *P* < 0.01, ***P < 0.001, ****P < 0.0001, n.s. represents *P* ≥ 0.05.

The morphology of cross sections of hydrogels is shown in Fig. 3a. The DAHA and DAHA/HAMA samples exhibit porous structures. In contrast, HAMA appears to have a non-porous structure. The porous network of DAHA and DAHA/HAMA is attributed to the instantaneous cohesion following dominant Fe^3+^-dopamine tris coordination, which induces inhomogeneity into the gel structure and produces denser, tris-complexed HA parts in the main gel network, in contrast to the looser pore parts. The average pore diameter in DAHA and HAMA/DAHA samples was 47.48 µm and 26.90 µm, respectively. Such pores are large enough to support cell trafficking, rapid nutrients and oxygen transport (Fig. 3b).

Previous research has demonstrated that the incorporation of porous structures into injectable hydrogels can promote medium perfusion, even in the absence of vascularization, and promote cell migration. Previous studies have developed in situ pore-forming hydrogels using various methods. However, these hydrogels have generally limited pore size, which can affect their permeability and performance. Attempts to enlarge the pores can weaken the hydrogel's mechanical strength, especially when exposed to the biomechanical stress present in dynamic organs or tissues. Taheri et al. fabricated injectable, pore-forming double-network chitosan hydrogels by employing a stepwise gelation and phase separation technique^8^. They obtained the average pore size of 10 µm which was larger that previous works and they could also address the inverse correlation between pore size and mechanical strength through a double-network configuration. The pore size obtained through Fe3 + -dopamine tris coordination in the current work is 2–3 times larger than what they achieved. A similar double-network approach was adopted to prevent deterioration of mechanical strength by the micropores.

The effects of Fe^3+^ concentration on the pore size was also evaluated and the results, shown in Figure S1, revealed that increasing the Fe^3+^ concentration from 10 to 20 mM led to a decrease in pore size within the hydrogel structure. This decrease can be attributed to the higher crosslinking density resulting from stronger coordination bonds between Fe^3+^ ions and dopamine groups. As the concentration of Fe^3+^ ions increased, more coordination bonds are formed, leading to a denser network with smaller pore size.

When the Fe^3+^ concentration was further increased from 20 to 25 mM, no significant change in pore size was observed. This suggests that maximum coordination interactions between Fe^3+^ ions and dopamine groups were achieved at concentrations above 20 mM, resulting in a saturation effect. Additional increases in Fe^3+^ concentration beyond this point did not lead to further changes in the porous structure.

#### Swelling measurements

The swelling properties of hydrogels were investigated by measuring weight changes of the hydrogels in PBS after 48 h of incubation. As shown in Fig. 3c, DAHA hydrogels exhibited a relatively high swelling capacity (38.36 ± 3.76%) which can be ascribed to its loser network and larger pores compared to other samples. A high swelling ratio may build up large pressures on the surrounding tissues, with detrimental effects. On the other hand, HAMA showed a relatively small swelling ratio of 10.52 ± 1.31%, which was attributed to its compact and non-porous structure and the hydrophobic nature of methacrylate groups. Hence, the combination of DAHA and HAMA yields a hydrogel with a viable degree of swelling (16.97 ± 2.62%).

Generally, hyaluronic acid-based hydrogels have been shown to have relatively high swelling ratios due to the hydrophilic nature of HA. A study conducted by Kim et al. reported a swelling ratio of approximately 300% after 24 h incubation in a phosphate-buffered saline solution at room temperature^36^. The swelling ratio of HA-based hydrogels can vary depending on a variety of factors, such as the degree of cross-linking and the presence of other components or functional groups in the hydrogel. Park et al. explored the relationship between the degree of crosslinking and the degree of acrylation of HA hydrogels and their corresponding swelling ratio^37^. Their findings revealed a decrease in swelling ratio as the degree of crosslinking or acrylation of the HA hydrogels was increased. The methacrylated HA synthesized and utilized to create highly crosslinked hydrogels in the current research, led to a reduction in the swelling ratio.

#### Enzymatic degradation

A degradation rate commensurate with wound healing or neo-tissue growth rate is desirable to promote tissue regeneration through replacement by newly regenerated tissue. The degradation rate of polymeric scaffolds strongly depends on material composition, including side groups, aromatic groups, and double or triple bonds, and polymer molecular architecture, including crosslinking and pore size.

Hyaluronic acid chains are broken by hyaluronidase. In previous studies, HA has been shown to degrade too fast to provide a sufficient support for tissue regeneration^25^. The degradation rate can be tuned by chemical modification and crosslinking methods^25^. Here, HA chains were modified with hydrophobic methacrylate side groups. These hydrophobic side groups together with the dual crosslinking mechanism contributed to the reduction of the degradation rate of the new hydrogel in the present work.

The degradation behavior of the hydrogels over a period of one month is shown in Fig. 3d. As observed, DAHA, HAMA, and HAMA/DAHA hydrogels exhibited a relatively fast, slow, and moderate rates of weight decrease and underwent a 75%, 47%, 62% loss of their initial weight within two weeks. This is because DAHA’s large pores and lose network led to accelerated degradation rate, while HAMA’s non-porous and dense network with hydrophobic methacrylate groups decreased the accessibility of hyaluronidase to the cleavage sites in the cross-linked hydrogel networks. This characteristic is consistent with the results related to the inner morphology and swelling behavior of the hydrogels.

Moreover, free dopamine has been reported to possess several biological properties, including antioxidant and anti-inflammatory effects, which can contribute to tissue protection and regeneration. Dopamine can also interact with specific cellular signaling pathways and receptors, modulating cellular responses, and promoting tissue repair. Additionally, the Fe^3+^ ions present in the complex may exert biological effects on cellular processes, such as oxygen transport, enzyme activity, and cell signaling. The specific biological response of released free dopamine and the dopamine-Fe^3+^ complex in the DAHA/HAMA hydrogel requires further investigation.

### Rheological and mechanical characterization

#### Compression test

Scaffolds provide mechanical and shape stability to the tissue defect. One important scaffold property is compressibility under stress. Scaffold failure under compression would hamper tissue repair. The mechanical properties of the biomaterials used for scaffolding, including toughness, should match those of the host tissue. Many studies of mechanobiology have highlighted the impact of mechanical properties of a scaffold on the seeded cells’ behavior. Human vocal folds undergo up to 30% strain. They have a Young’s modulus around 20 kPa at 30% strain.

**Figure 4:**
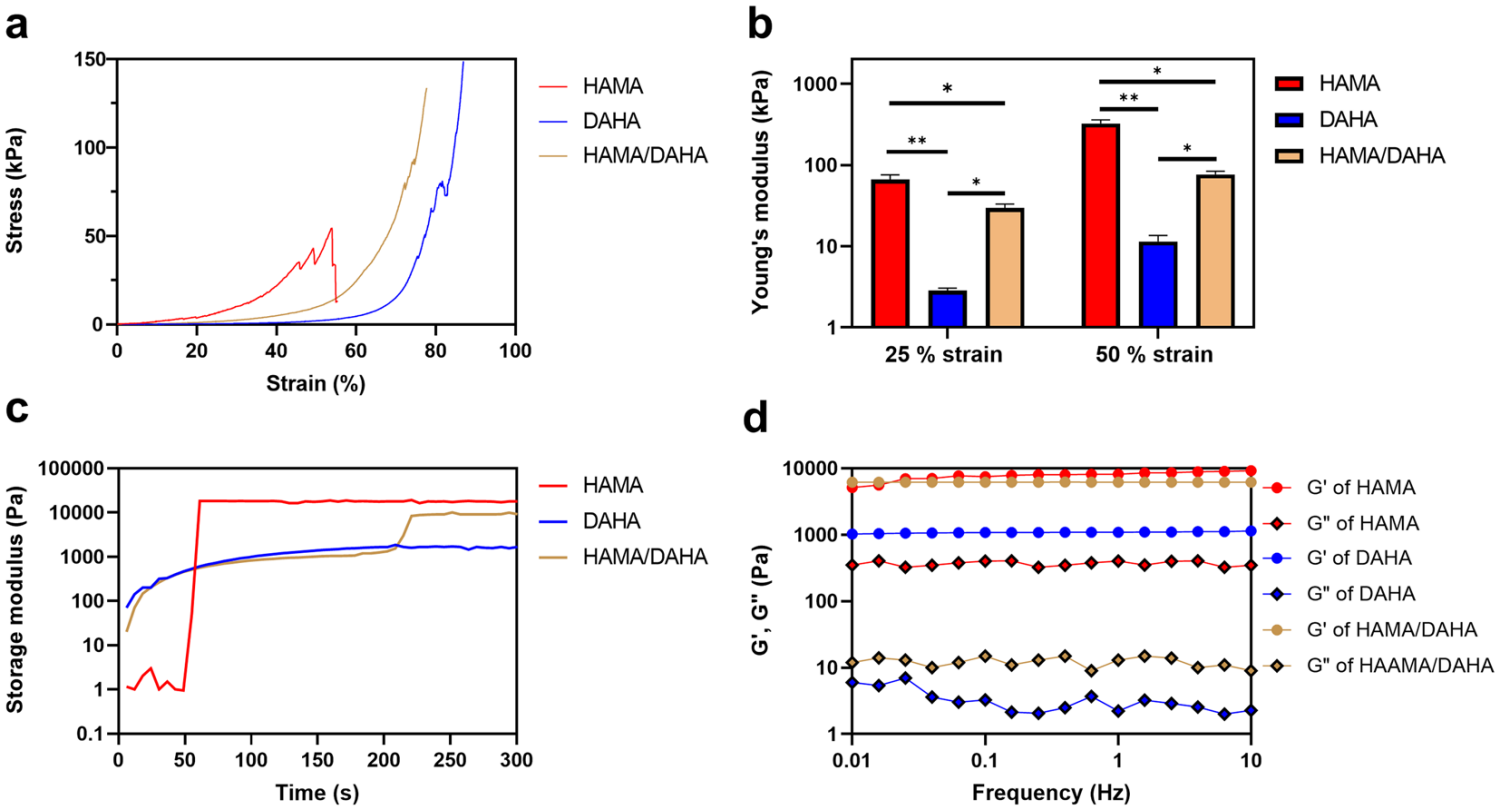
Mechanical and viscoelastic properties of hydrogels. (**a**) Compression test results at 1 mm/min displacement rate. (**b**) Calculated young’s modulus at 25% and 50% strains. (**c**) Gelation kinetics of samples at 0.1% strain and 1 Hz frequency. (**d**) Frequency sweep dynamic rheological data for different samples. Sample size, *N* = 3; ** represents *P* < 0.01, *P < 0.05, n.s. represents *P* ≥ 0.05.

As shown in Fig. 4a,b, HAMA hydrogels display a brittle network with a relatively high compressive modulus of 66.5 kPa at 25% strain, which is due to the covalent crosslinking and a loss of mobility of the entanglements of chains. On the other hand, DAHA samples had a low compressive modulus of 2.8 kPa at 25% strain, which can be attributed to the Fe^3+^-dopamine physical coordination. Hence, the combination of HAMA and DAHA yields a double network hydrogel with optimal compressive modulus of 29.9 kPa at 25% strain, and flexibility for vocal fold tissue engineering^25^.

#### Gelation time

A controllable gelation speed is important for injectable hydrogels, especially for hydrogels to be formed in situ in wet conditions. The gelation speed must be fast enough to prevent leakage to the surrounding area, but slow enough to prevent needle clogging.

The hydrogels’ gelation kinetics were investigated using a time sweep mode. All samples exhibited a fast sol–gel transition, as shown in Fig. 4c. The DAHA samples jellified in around 6 s and reached a final storage modulus of 1.66 kPa. The HAMA samples solidified upon UV exposure and reached a final storage modulus of 17.9 kPa. The DAHA/HAMA hydrogels displayed a viable gelation kinetics for in situ injection applications. This is because their first network, which is a physical crosslinking induced by Fe^3+^-catechol coordination, is rapidly formed in 6 s, which is fast enough for in situ injection purposes. This lose network is then strengthened by a chemical crosslinking between methacrylate groups upon UV exposure.

#### Viscoelastic properties

The viscoelastic properties of vocal fold tissue are essential for efficient phonation and the ability to produce a range of pitches and volumes. Any abnormalities in the viscoelastic properties of the tissue can lead to voice and/or speech disorders. The storage (G’) and loss (G”) moduli were measured as a function of frequency to provide information about the damping properties of the tissue. The storage and loss moduli of human vocal fold tissue have been reported to range from 1–10 kPa and 10–30 Pa, respectively. These values suggest that vocal fold tissue is relatively soft and compliant, with a nearly critical damping to prevent damage during phonation^38^.

To elucidate the viscoelastic properties of the hydrogels, frequency sweep experiments were carried out. A high G’ in comparison to G” indicates strong intermolecular interactions. This results from the ability to resist intermolecular slippage due to the relative strength of the loosely associated gel structure occurring in the three-dimensional network. This test also provides insight into the mechanisms at work during injection. At low frequencies, the strain simulates a disturbance during loading or storage. At higher frequencies, the tests may provide information about properties during injection. As observed in Fig. 4d, all samples behaved consistently as perfectly elastic gels, with G′ > G″. The storage modulus exhibits nearly a frequency independent trend, which indicates the formation of stable crosslinked networks. As expected, the G’ and G” values of HAMA are much greater than those of DAHA, especially for G’, demonstrating the fact that in HAMA network polymer chains are crosslinked with the irreversible covalent bonds as apposed to DAHA’s reversible and lose network. The optimal elasticity and viscosity of HAMA/DAHA is important for adhesion to soft tissues, which can better bear load and dissipate elastic energy by viscous deformation and keep in harmony with flexible soft tissues. The measured values of storage and loss moduli of HAMA/DAHA samples match those of native VF tissue, as required for VF regeneration.

#### Yield point

The hydrogels’ linear viscoelastic region (LVER) and yielding point were measured using strain sweep test. Yield stress/strain is defined as the stress or strain below which no flow occurs while above this critical point, the structure of the hydrogels breaks down and the values for G’ and G” became highly strain sensitive.

**Figure 5:**
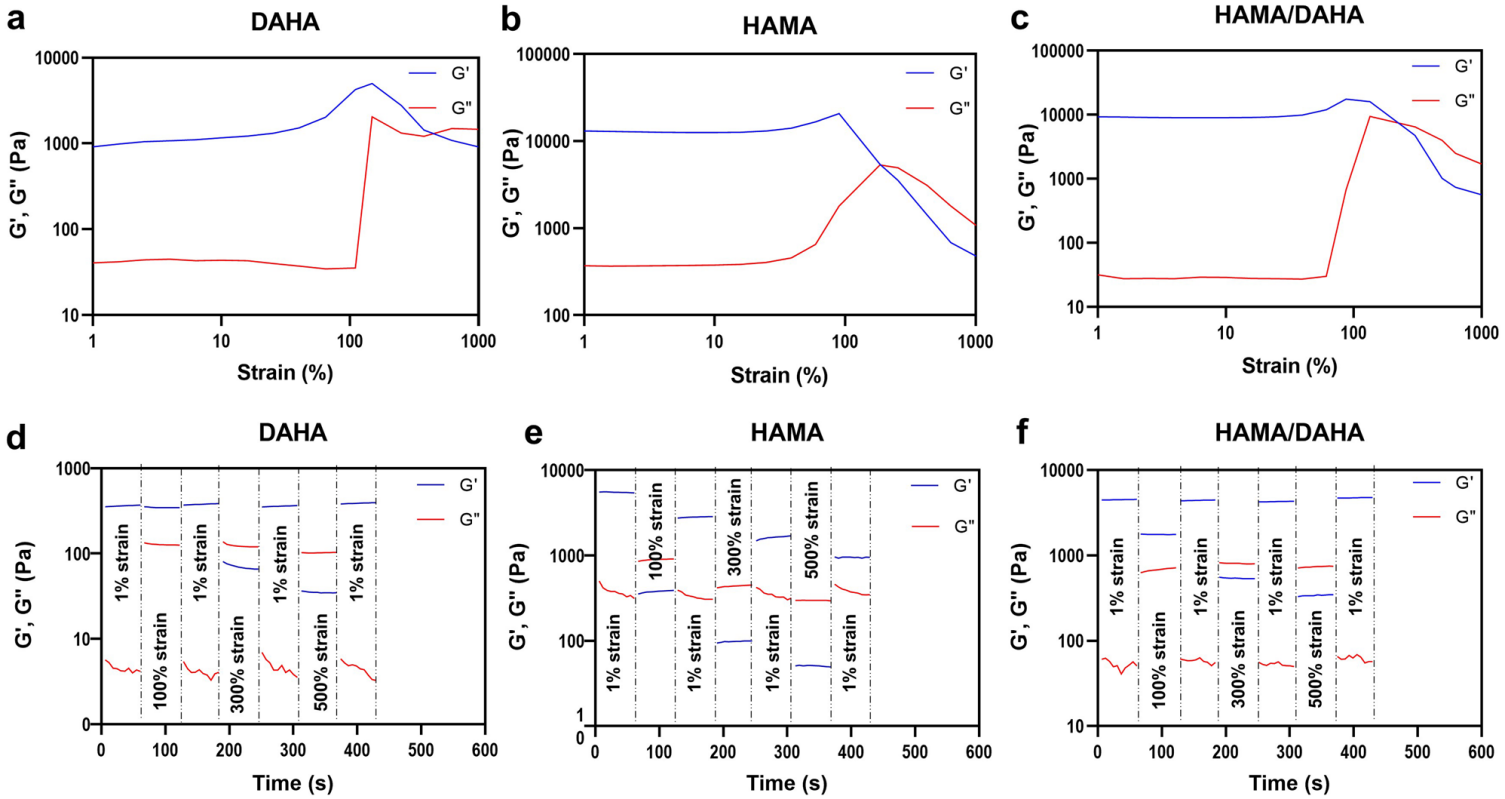
Rheological measurements showing the breaking point and self-healing property of hydrogels. (**a–c**) Strain-sweep tests revealing samples’ yield strain. (**d–f**) self-healing behavior of samples through the alternative application of low (1%) and high (100%, 300%, and 500%) strain cycles.

As shown in Fig. 5a–c, a solid–liquid transition, indicated by the G’ and G” crossover, tend to occur at higher strains for DAHA and DAHA/HAMA compared to HAMA. This is consistent with DAHA’ flexible and HAMA’s brittle network. This will because of the HAMA’s covalent irreversible and dense crosslinking as apposed to DAHA’s physical and reversible crosslinking.

#### Self healing

The self-healing behavior of hydrogels was investigated using a combination of oscillatory strain sweeps and time sweeps, Fig. 5d–f. As shown in Fig. 5a,b, DAHA and DAHA/HAMA hydrogels were degraded under increasing oscillatory strain, which induces moduli inversion at each crossover point. However, time sweep results indicate that hydrogels exhibit instantaneous healing and recover their original strength after removal of the stress. Such self-healing behavior of the gel is attributed to interactions between Fe^3+^ and dopamine groups. The coordinate bond plays a sacrificial role under high stress, while gels are degraded by shear strain. However, the reversible and dynamic equilibrium of coordinate bonds readily reorganizes the gel network, leading to the rapid re-formation of gels. On the other hand, Fig. 5f, clearly demonstrates that the HAMA formulation lacks self-healing properties. After subjecting the gel to high strains and causing its fracture, the HAMA hydrogel failed to restore its original storage modulus. This irreversible behavior can be attributed to the presence of irreversible chemical crosslinking between the methacrylate groups in HAMA as apposed to the reversible dopamine-iron crosslinking.

To further evaluate the self-healing ability of HAMA/DAHA hydrogel, a macroscopic self healing test was also performed. As shown in Figure S2. the hydrogel can heal and restore its initial integrity after cutting and recombining. This emphasizes on the dynamic and reversible nature of Fe^3+^-dopamine coordination.

### Biological characterization

#### Tissue adhesion study

**Figure 6:**
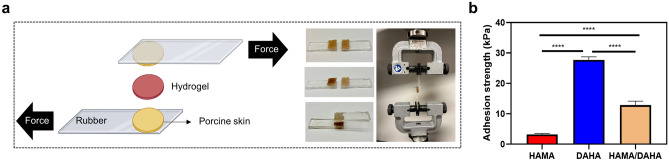
Tissue adhesion property. (**a**) Schematic of adhesion measurement. (**b**) Calculated hydrogels’ adhesion strength to porcine skin. Sample size, *N* = 3; ** represents *P* < 0.01, ***P < 0.001, ****P < 0.0001, n.s. represents *P* ≥ 0.05.

Lap shear tests were performed to assess the tissue adhesive properties of hydrogels. The adhesion strength was determined at the point of maximum stress. As shown in Fig. 6, the HAMA hydrogels showed negligible adhesion on porcine skin (3.2 ± 0.3 kPa), while the DAHA and DAHA/HAMA hydrogels showed significantly enhanced adhesion strength (27.7 and 12.8 kPa, respectively). Although the obtained adhesion strengths are much lower than these for strong adhesives, such as chitosan-based hydrogels, they are comparable to those of commercially available fibrin glues (16.6 kPa) and significantly higher than non-dopaminated HA hydrogels.

#### Cytotoxicity

**Figure 7:**
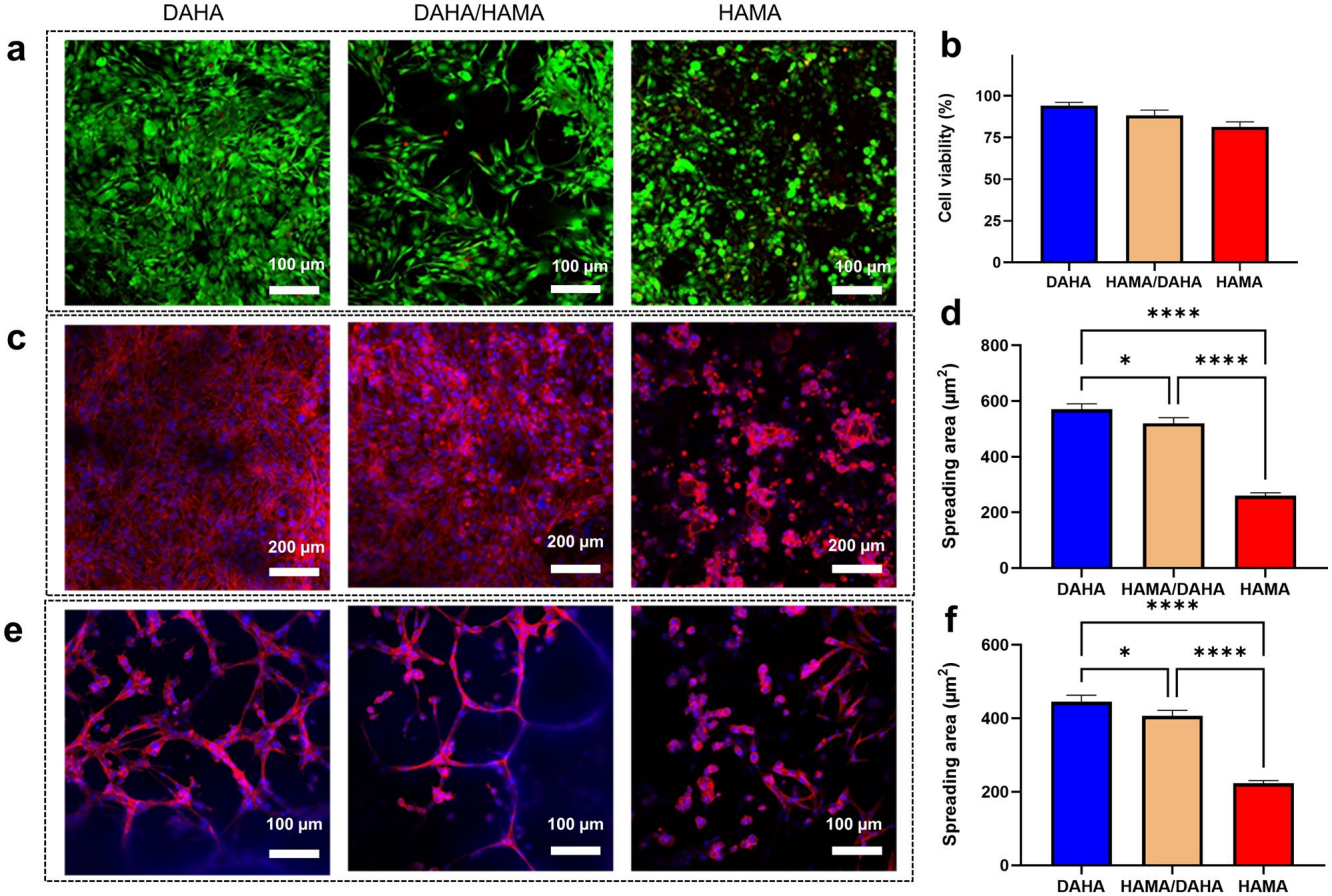
Viability and morphology of hVFFs cultured in different hydrogels at day 7. (**a**) Confocal images of live/dead cells seeded on hydrogels; live cells are shown in green and dead cells in red. (**b**) Cell viability percentage. (**c**) Confocal images showing the morphology of cells cultured on the surface of samples. (**d**) Projected cell spreading area on the samples. (**e**) Confocal images showing the morphology of cells cultured inside the samples. (**f**) Projected cell spreading area within the samples.

The cytotoxicity of hydrogels was examined by fluorescence staining with a Live/Dead assay on day 7. Live cells and dead cells were fluorescently labeled green and red, respectively, as shown in Fig. 7a. The detection of live/dead cells was performed to quantify the viability of fibroblasts seeded on hydrogels after culture for 7 days. As shown in Fig. 7b, the viability of cells in DAHA, DAHA/HAMA, and HAMA samples was calculated to be 93%, 86%, and 78%, respectively. Viability was thus deemed satisfactory. Although the HAMA group had a higher number of dead cells than the other groups, the number of dead cells was minimal when compared to the number of live cells.

To assess the cytotoxicity of the degradation byproducts, WST-1 assay was performed on the degradation medium at predetermined time points. The results, displayed in Figure S3, showed that the degradation byproducts of the hydrogel did not exhibit significant cytotoxic effects. Further comprehensive biocompatibility studies, including in vitro and in vivo assessments, are necessary to fully evaluate the safety profile of the hydrogel.

#### Cell adhesion

In vitro cell attachment and cytoskeletal morphology after 7 days were assessed using rhodamine–phalloidin staining for actin cytoskeleton and Hoechst 33,258 staining for nuclei followed by confocal microscopy. As shown in Fig. 7c,e, cells were stacked onto each other to form cell clusters on HAMA samples. This is because HA lacked cell adhesion motifs, resulting in poor cell adhesion. The addition of dopamine markedly enhanced cell adhesion. Cells exhibited a healthy and fibroblast-like morphology on DAHA and DAHA/HAMA hydrogels. This presumably attributed to the interaction between dopamine groups and integrin receptors present on the cell membrane. Comparatively, reduced actin and weaker fluorescence were observed in the DAHA/HAMA group than in the DAHA group. To further assess the effects of hydrogel composition on cell morphology, a quantitative analysis of cell spreading was performed^39^. The projected cell surface area was estimated using image analysis software. As shown in Fig. 7d,f, the cell spreading area on top of DAHA, DAHA/HAMA, and HAMA was calculated to be 570 µm^2^, 525 µm^2^, and 263 µm^2^, respectively. Moreover, the cell spreading area inside the hydrogels was calculated to be 435 µm^2^, 408 µm^2^, and 224 µm^2^ for DAHA, DAHA/HAMA, and HAMA, respectively.

## Conclusion 

In this work, a novel method was used to prepare a double network HA-based hydrogel using two modified versions of HA, including dopamine-grafted HA and methacrylated HA, as an injectable scaffold for vocal fold tissue engineering applications. The double network hydrogel was obtained via the Fe^3+^-dopamine coordination complexation (instantaneous physical crosslinking) and the photo-crosslinking of methacrylate groups (UV-initiated chemical crosslinking). The initial fast gelation (< 6 s) makes hydrogels suitable candidates as in situ forming scaffolds. The hydrogels also exhibited strong tissue adhesion, thanks to grafted dopamine groups, as well as a microporous structure with self-healing property, which are both attributed to Fe^3+^-dopamine coordination. A microporous structure is very beneficial for cell ingrowth. The self healing property is a valuable feature for load bearing tissues, such as human vocal folds. Furthermore, the chemical crosslinking between methacrylate groups enhances mechanical properties, decreases swelling ratio, and slows degradation. The DAHA/HAMA hydrogel demonstrates a unique combination of mechanical, structural, and biological properties*,* making it a promising candidate for in situ repair of vocal folds*.* This injectable hydrogel offers cytocompatibility, porosity, adhesiveness, and enhanced mechanical properties, distinguishing it from existing injectable hydrogels. Further investigations involving comprehensive cell and animal studies are needed for the translation of the DAHA/HAMA hydrogel into clinical use. These additional studies will provide valuable insights into its safety, efficacy, and long-term performance, thereby advancing its progress towards clinical application.

## Materials and methods

### Materials

Chemicals used in the current work were purchased from Sigma–Aldrich and used without further purification unless otherwise stated.

### Polymer modifications

Two different derivatives of HA, namely hyaluronic acid methacrylate (HAMA) and dopamine grafted aldehyde-hyaluronic acid (DAHA) were obtained through following polymer modification procedures.

#### Synthesis of HAMA

Hyaluronic acid was methacrylated according to the procedure described by Bencherif et al.^40^. In short, HA (1.0 g) was first dissolved in 200 mL phosphate buffer saline (PBS, pH 7.4) and 67 mL of dimethylformamide (DMF); then, 13.3 g of methacrylic anhydride and 6.7 g of triethylamine were added to the HA solution. After 5 days reaction, the solution was dialyzed against deionized water for 3 days, followed by lyophilization for another 3 days. The lyophilized HAMA was analyzed by proton Nuclear Magnetic Resonance (^1^H NMR) spectroscopy. The degree of methacrylation was determined by normalizing the peaks at a chemical shift of 6 ppm, representing the methacrylate group, and by peaks between 3 and 4 ppm, which represent native HA.

#### Synthesis of DAHA

Aldehyde-modified hyaluronic acid (AHA) was synthesized following a previously described method^15^. In brief, 1.0 g of HA was dissolved in 100 mL deionized water; next, 0.5 g of sodium periodate was added to the solution. After 5 h of mixing in dark conditions, the reaction was quenched by adding 1 mL ethylene glycol to eliminate the excess periodate. To remove the unreacted agents, the reaction solution was exhaustively dialyzed against deionized water followed by lyophilization to obtain the oxidized polymer.

Dopamine was grafted onto AHA through a Schiff base reaction between the amino groups in dopamine and aldehyde groups in AHA^15^. Specifically, AHA (1 g) was dissolved in 100 mL of phosphate buffered saline (PBS, pH = 5.0) after which 1 g of dopamine hydrochloride was added and mixed for 10 h at room temperature. The functionalization process was finalized by introducing 50 mg of sodium borohydride to the reaction followed by further stirring for 30 min. Sodium borohydride is a reducing agent which converts the reversible double bond in the Schiff's base into a stable single bond. This modification enhances the stability of the bond between dopamine and aldehyde, preventing its premature degradation and ensuring its long-term structural integrity. The reaction solution was then dialyzed against deionized water for 3 days and lyophilized for an additional 3 days to obtain DAHA. Grafting of dopamine onto AHA was confirmed by Fourier-transform infrared (FT-IR) spectroscopy based on the changes in the vibrational absorption peaks. The degree of substitution of dopamine (DS) was determined from the proton NMR spectrum.

### Hydrogel fabrication

To form a hybrid hydrogel, HAMA and DAHA macromers were separately dissolved in deionized water at 0.6% w/v and 5% w/v, respectively and mixed with an equal volume. FeCl_3_ and LAP were then added to the mixture at the final concentration of 20 mM and 0.25% w/v, respectively. After mixing for a few seconds, the pregel was exposed to UV light (365 nm; 36 mW/cm^2^) for 30 s.

### Scanning electron microscopy

The morphology of the hydrogels was investigated using scanning electron microscopy (SEM). The hydrogels were dried using critical point drying (CPD) in order to maintain its structure. Then, dried samples were cross-sectioned and sputter-coated with gold, before their surface morphology was monitored by SEM instrument. The SEM images were then analyzed using the Image J software to measure the pore size.

### Swelling measurements

To assess the equilibrium swelling ratio, freshly prepared hydrogels were weighted (W_i_), and then immersed in PBS under agitation via an orbital shaker at 75 rpm and 37 °C. After 48 h, the excess of water on the surfaces of gel was absorbed and the swollen hydrogel was immediately weighed (W_s_). The swelling ratio of the hydrogel was determined based on the sample weight increase percentage using the formula (*W*_*s*_ − *W*_*i*_)/*W*_*i*_ × 100%.

### Rheological and mechanical measurements

#### Compression test

Mechanical characterization was carried out using a Instron 3360 electronic universal testing machine (Instron corporation, MA, USA). The compression test was operated at a constant speed of 1 mm min^−1^, on cylindrical shaped hydrogels with a height of 5 mm and diameter of 10 mm. The samples’ compressive modulus was determined from the slope of the stress vs. strain relation. The compression modulus of the samples was calculated for 25% and 50% strain amplitudes.

#### Gelation time

The gel-forming kinetics were studied using a TA Instruments photorheometer at the oscillatory time sweep mode at 1 Hz and 0.1% strain. The gelation point was determined as the cross-over point of the storage (G′) and loss modulus (G″).

#### Yield point

Amplitude sweep tests from 1% to 1,000% strain at the frequency of 1 Hz were conducted using the photorheometer to find the linear viscoelastic region (LVER) and also the yielding point of the hydrogels. The G’/G’’ cross-over point was evaluated from amplitude sweeps to estimate the brittleness of hydrogels.

#### Self-healing

The self-healing property of the hydrogels was evaluated by visual inspection and rheology assays. For visual evaluation, a hydrogel disk was cut into pieces using a razor blade. The gel pieces were then reassembled and allowed to self-heal for 10 min at room temperature without additional treatment. An image of the integrated gel disk was then taken. To evaluate this property using rheology tests, amplitude sweeps were carried out in the strain range 1–1,000% to determine the rupture strains of the hydrogels. Then the gel re-formation was identified by successive time sweeps where the strain varied from low (1%) to high (100, 300, and 500%) values.

#### Viscoelastic properties

To evaluate the dynamic viscoelastic properties of samples, frequency sweep measurements were carried out over the range of frequencies from 0.1 to 10 Hz, at 0.1% strain using the photorheometer.

### In vitro degradation test

Freshly prepared samples were rinsed in hyaluronidase solution (100 U ml^−1^, dissolved in PBS)) and incubated at 37 °C on a rotary shaker (150 rpm) for in vitro degradation test. At the set time point, three samples were extracted and weighed (Wt) after wiping off the solution on the surfaces. The remaining weight fraction was determined as (Wt/Wi) × 100 and plotted against time^41^.

### Tissue adhesion test

The adhesion of the hydrogels was evaluated through lap shear tests using porcine skin as the tissue model^42^. To this end, two pieces of rubber were overlapped over a span of 10 mm between which 500 µl of the pregel was spread, and the area of bonding was 20 mm × 10 mm. This was followed by irradiation with UV light at 36 mW/cm^2^ intensity for 10 min. The rubbers were then tested for tensile shear strength using an Instron testing machine at 10 mm min^−1^ crosshead speed.

### Cellular evaluations

Immortalized human vocal fold fibroblasts (hVFFs) at passage 8 were cultured in DMEM medium supplemented with 10% fetal bovine serum and 1% penicillin–streptomycin. Culture was maintained at 37 °C in a humid atmosphere containing 5% CO_2_. The media were changed every 3 days for 2D cultures. When cells reached 80% confluence, they were disassociated using 0.25% trypsin containing 1 × 10 ^3^ M ethylenediaminetetraacetic acid (EDTA).

#### Cell viability

The cytocompatibility of the samples was evaluated using a live/dead assay. Briefly, hVFFs were suspended in the pregel at the final cellular concentration of 1 million mL^−1^ and the mixtures were then injected into ibidi chamber slides to form hydrogels. DMEM medium supplemented with 10% FBS and 1% penicillin was used for culturing the cells in the incubator. After 3 days incubation, the medium was removed and 200 µl of DMEM supplemented with 0.1 µl of calcein-AM and 0.4 µl of ethidium homodimer was added into each well, followed by 30 min of incubation in darkness. The stained cells were imaged using a confocal laser scanning microscope (LSM710).

#### Cell adhesion

To assess the morphology and attachment of hVFFs to hydrogels, the cell-laden samples were first washed three times with PBS and then fixed using 4% paraformaldehyde for 20 min at room temperature. Cells were then permeabilized with 0.1% Triton-100 for 5 min, and subsequently washed twice with PBS. Cells were then incubated in 1% BSA solution in PBS for 30 min. Finally, the cell nuclei and F-actin were stained with Alexa Fluor 633 phalloidin and Hoechst 33,258, respectively. In brief, phalloidin was dissolved in a 1% BSA solution at a final concentration of 5 U/ml, added to cell-laden hydrogels, and incubated for 20 min. The cells were then washed twice with PBS and stained with Hoechst (1 μg/mL) for 10 min. The nuclear stain solution was removed and replaced with PBS for visualization using a confocal laser scanning microscope (LSM710).

### Supplementary Information

Supplementary Figures.

## Data Availability

The data that support the findings of this study are available from the corresponding author, upon reasonable request.
