# Supplementary material for: Injectable, pore-forming, self-healing, and adhesive hyaluronan hydrogels for soft tissue engineering applications
Source: Sci Rep. 2023 Aug 31;13:14303. doi: 10.1038/s41598-023-41468-9 (PMC10471737; doi:10.1038/s41598-023-41468-9)
Supplement: Supplementary file 1 — Supplementary Figures. [file 41598_2023_41468_MOESM1_ESM.pdf]

Supplementary information

Injectable, pore-forming, self-healing, and adhesive hyaluronan hydrogels for soft tissue engineering applications

Sara Nejati, Luc Mongeau*

Department of Mechanical Engineering, McGill University, Montreal, Canada

* Corresponding author E-mail: luc.mongeau@mcgill.ca

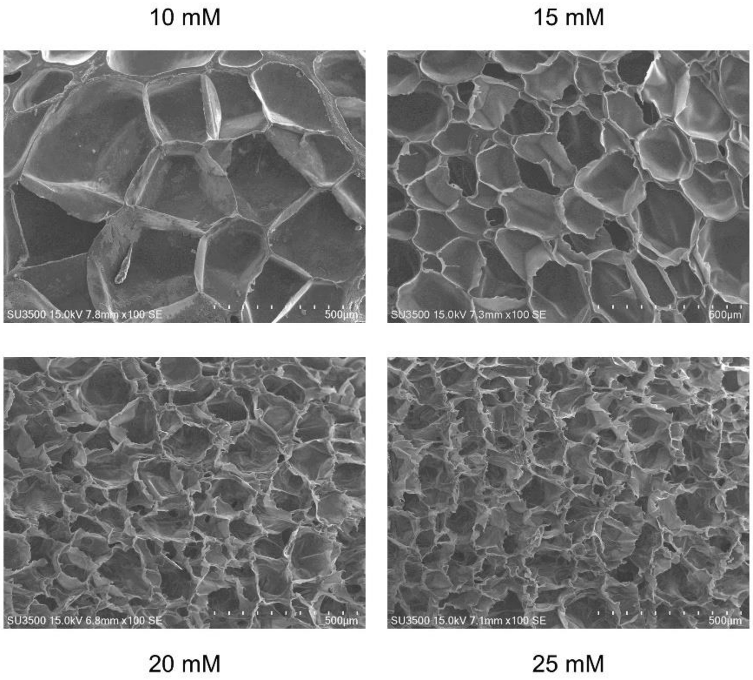

Figure S1. SEM images of DAHA hydrogels prepared using varying concentrations of Fe^{3+}.

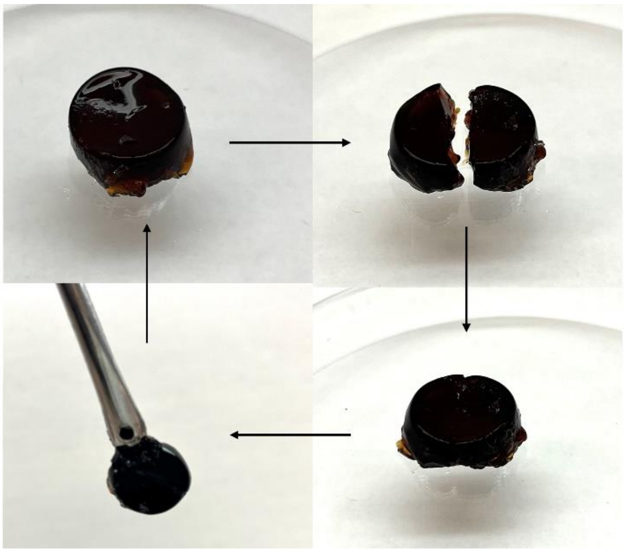

Figure S2. Photographs of the self-healing ability of DAHA/HAMA hydrogel.

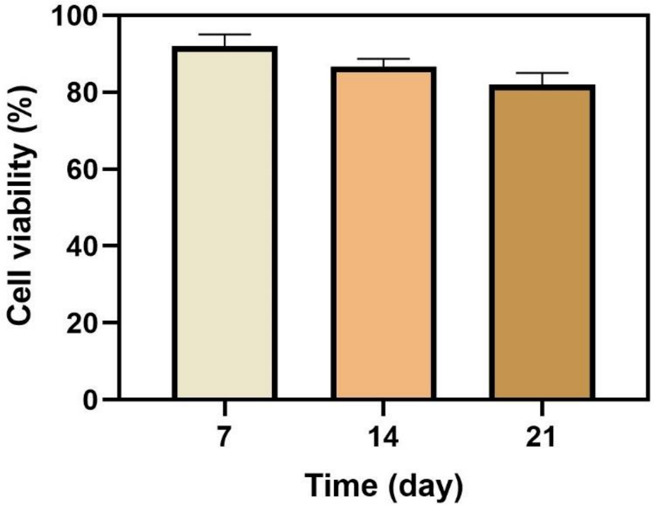

Figure S3. WST-1 assay showing the viability of hVFFs cultured with DAHA/HAMA degradation medium at predetermined time points.
